# Non-death loss and GenAI: ethical reflections on griefbots of the living

**DOI:** 10.3389/fgene.2026.1825398

**Published:** 2026-04-30

**Authors:** Alexander Manevich, Yuval Haber

**Affiliations:** 1 Department of Clinical Psychology of Adulthood and Aging, Ruppin Academic Center, Kfar Monash, Hefer Valley, Israel; 2 School of Psychological Sciences, Faculty of Social Sciences, International Laboratory for the Study of Loss, Bereavement and Human Resilience, University of Haifa, Haifa, Israel; 3 The Program of Hermeneutics and Culture, Interdisciplinary Studies Unit, Bar-Ilan University, Ramat Gan, Israel

**Keywords:** non-death loss, generative artificial intelligence (GenAI), biomedical ethics, dementia, griefbots, PremorbidBot, ALIVE model

## Abstract

The rapid integration of artificial intelligence into loss and bereavement marks a paradigmatic shift in the way humans maintain the relationship with the dead. Rather than relying primarily on memory, individuals may now engage with algorithmic entities that simulate the deceased by drawing on personal data to reproduce patterns of language, voice, and behavior. Although such technologies may respond to the enduring human need for ongoing dialogue with the deceased, they raise significant questions about the boundaries between life and death, as well as about their potential impact on the grieving process. To date, scholarly attention has focused almost exclusively on bereavement following death. However, it is reasonable to anticipate that these applications will soon extend to forms of loss that do not result from death, particularly situations in which individuals grieve loved ones who remain alive. Introducing the concept of a *PremorbidBot*, a digital representation based on the individual’s premorbid personality, this article examines the ethical implications of such developments in advance of their wider adoption. The analysis proceeds through the prism of Beauchamp and Childress’s four principles of biomedical ethics, with dementia serving as an illustrative case, given its unique convergence of physical presence and progressive psychological absence. Finally, we propose the ALIVE model (Autonomy & Consent, Living Presence, Intended Benefit, Vigilance Against Harm, Equity & Accountability) as a preliminary framework for ethical evaluation and decision-making in AI-mediated non-death loss contexts, a conceptual foundation rather than a fully elaborated model, with operationalization left to future empirical and normative inquiry.

## Introduction

Traditionally, mourning entailed transforming the relationship with the deceased from an external bond into an internalized one, grounded in memories, interpretations, and internal representations, enabling an ongoing sense of connection and emotional accompaniment throughout life (Klass and Steffen, 2018). With the integration of artificial intelligence (AI) into the domains of loss and bereavement, new possibilities are emerging that may alter this process. Rather than relying solely on memory, individuals may now encounter algorithmic entities capable of simulating language, voice, and behavioral patterns of the deceased. These applications, sometimes referred to as *deathbots, thanabots,* or *griefbots,* generate a novel form of digital presence that emulates the person who is no longer alive (Brescó de Luna and Jiménez-Alonso, 2024).

Although such technologies may respond to the profound human need for dialogue with the deceased, their use raises substantial concerns regarding the boundaries between authentic memory and computational representation. A central question arises as to whether these technologies constitute a legitimate continuation of human bonds through new means that offer comfort, or whether they risk creating an illusion that destabilizes traditional distinctions between life and death and may cause significant harm to the grieving process (Manevich et al., 2025). The answer may not be binary and is likely to depend on context, individual circumstances, and the manner in which these technologies are implemented. What is well established is that such technologies exist and are in active use; what remains less certain is how they reshape the experience of grief over time.

To date, both technological applications and academic scholarship in this field focus almost exclusively on the role and implications of AI among individuals bereaved by death (e.g., Christensen and Sumiala, 2024; Degni, 2025; Dwi, 2025; Fabry and Alfano, 2024; Hollanek and Nowaczyk-Basińska, 2024; Hurtado Hurtado, 2023; Krueger and Osler, 2022; Manevich and Aluma, 2025; Morris and Brubaker, 2025; O’Connor, 2024; Pizzoli et al., 2024; Recuber, 2023; Seckman, 2025; Yang and Khanna, 2025). However, given the rapid development of these technologies and their expanding use among bereaved populations, it appears likely that their application will soon extend to contexts of non-death loss; that is, situations in which individuals grieve loved ones who remain alive due to conditions such as neurodegenerative disease, traumatic brain injury, or prolonged disappearance. This extension, while not yet documented, represents a plausible near-term development given current trajectories of technological adoption and the broadening scope of AI applications.

This anticipated expansion calls for a proactive ethical discussion that places the wellbeing of both users and represented individuals at its center. Yet to date, no such discussion has been undertaken. The present article seeks to fill this gap by offering what is, to the best of our knowledge, the first theoretical and ethical framework for examining the use of *griefbot* technologies in contexts of non-death loss, where the represented individual remains alive. Because this analysis is anticipatory by design, it distinguishes between three epistemic registers: findings documented in the existing literature on grief technologies following death, plausible near-term extensions of these findings to non-death loss contexts, and considerations that remain hypothetical, requiring future empirical investigation.

The article is structured as follows. It opens with a brief overview of the integration of AI into loss and bereavement. It then introduces the field of non-death loss and its distinctive characteristics. Next, the central ethical issues arising from the use of AI technologies in situations of dementia are examined through the four principles of biomedical ethics articulated by Beauchamp and Childress (1979). The article concludes with a summary of the main arguments, briefly introduces the ALIVE model as a preliminary framework for ethical evaluation and decision-making in AI-mediated non-death loss contexts, and proposes directions for future reflection and research.

## The Interface between artificial intelligence and loss and bereavement

### Bereavement after death

Generative artificial intelligence (GenAI) technology represents a significant development in human history, with growing implications for mental health assessment and treatment (e.g., Bhatt, 2025; Blease and Rodman, 2024; Cruz-Gonzalez et al., 2025; Haber et al., 2024; Olawade et al., 2024). Perhaps most significantly, through the mediation of language, AI introduces the possibility of humans coming to know themselves and their environment through the mirror of a non-human other (Haber et al., 2024; Vallor, 2024). Recent findings suggest that general-purpose chatbots such as *ChatGPT* and *Claude* are rapidly becoming among the largest *de facto* mental health service providers worldwide, as a growing proportion of users turn to them for emotional support, relationship guidance, and psychological needs (e.g., Cross et al., 2024). This rapid and largely unregulated adoption carries considerable implications. Such chatbots may expand access and availability of mental health support, yet they also raise concerns regarding emotional dependency, algorithmic bias, data privacy, and the erosion of authentic human connection (Elyoseph et al., 2024).

Among the less explored applications of AI in mental health is its potential to reshape the ways in which individuals experience and process loss and death. One notable manifestation of this potential is the emerging field of “grief tech”, which may substantially transform the ways in which individuals experience loss, manage it, and commemorate their loved ones. These technologies reflect a deep human longing for continued connection, even if symbolic or simulated, with those who are no longer present. In doing so, they participate in a broader cultural tradition of sustaining ongoing bonds with the dead and mediating their presence in the lives of the living (Manevich et al., 2025).

This field encompasses a wide range of applications (Hollanek and Nowaczyk-Basińska, 2024). At one end are bots designed to simulate a specific deceased individual by processing personal data and reconstructing conversational patterns. At the other end there are more general systems that function as supportive companions and provide emotional responses to bereaved users without reference to a particular person. A further distinction exists between generative systems, which produce new content based on learned patterns, and retrieval-based systems, which are limited to presenting preexisting content. This distinction is not merely technical but experiential and ethical, as generative technologies carry different implications for grief processing, perceived presence, and emotional engagement. For instance, a generative bot that produces novel utterances in the voice of the deceased may foster a stronger sense of presence, but also a greater risk of misrepresentation.

Nevertheless, the limited evidence available suggests that applications simulating the deceased may serve a supportive role in coping with loss, potentially functioning as *transitional objects* in the Winnicottian sense (1971). Building on this notion, Haber et al. (2025), in a theoretical and ethical analysis, proposed that GenAI may more broadly function as a potential space for the externalization of internal experiences, enabling their visual and dialogical exploration within a therapeutic context. Although that framework does not directly address bereavement, it provides a theoretical foundation for understanding how AI-mediated interaction may facilitate the expression of self-parts, a capacity that is potentially relevant to the processing of loss. Taken together, these theoretical frameworks suggest that a griefbot, while differing from a traditional transitional object in that it actively responds and generates content, may nonetheless create an intermediate space in which a symbolic bond mediates between physical absence and psychological presence.

Preliminary empirical findings lend some support to this theoretical framing. For example, in a qualitative study, Xygkou et al. (2023) found that bereaved participants described conversations with griefbots as a safe space for emotional expression and as a means of coping with loneliness. Some reported that the interaction felt more comfortable and open than conversations with formal therapists or close family members. Importantly, participants did not perceive the bots as substitutes for human relationships but rather as complementary and temporary tools that facilitated internal processing of loss and preserved a sense of connection with the deceased. Similarly, Fu et al. (2025) found that bereaved family members of cancer patients expressed cautious openness toward AI-based mourning technologies, with acceptance shaped by both grief perception and ethical concerns.

At the same time, emerging concerns point to potential risks associated with the use of these technologies. In certain cases, what is intended to function as a supportive tool in the grieving process may paradoxically intensify and prolong the pain of loss. Scholars have warned that such technologies may disrupt the grieving process by delaying acceptance of the finality of the loss, and that without adequate safeguards they risk causing significant psychological harm to vulnerable users. Moreover, there is a risk of emotional dependency and even compulsive patterns of use, particularly when systems are continuously available and provide immediate responses to needs for closeness. In such circumstances, users may come to prefer interaction with the bot over engagement in real human relationships, potentially undermining the development of internal coping mechanisms and long-term adaptation to loss (e.g., Hollanek and Nowaczyk-Basińska, 2024; Manevich and Aluma, 2025).

These concerns find additional grounding in neurobiological research on prolonged grief disorder (PGD) (American Psychiatric Association, 2022; World Health Organization; World Health Organization, 2021), which has been conceptualized as a condition with addiction-like characteristics in terms of the underlying neural mechanisms involved (Kakarala et al., 2020). It is plausible, though not yet empirically established, that repeated interaction with a digital representation of the deceased may activate similar mechanisms and reinforce persistent attachment that impedes processing and adaptation. Some empirical support for this concern has begun to emerge. Kawashima et al. (2023) found that the desire to maintain a digital bond with the deceased significantly predicted higher levels of PGD approximately 5 months after baseline measurement. Additionally, it has been suggested that the use of such technologies may give rise to new forms of psychopathology that have not yet been identified or defined within existing psychiatric diagnostic systems (Gilat, 2025). These concerns are echoed in professional discourse: Yockel et al. (2025) found that clinicians expressed significant reservations regarding the introduction of AI tools to children anticipating parental loss, emphasizing the need for developmental sensitivity and professional oversight.

Despite growing scholarly attention to the ethical, philosophical, and socio-technical dimensions of AI-mediated grief technologies (e.g., Campbell et al., 2025; Elder, 2019; Figueroa-Torres, 2024; Grandinetti et al., 2020; Hollanek and Nowaczyk-Basińska, 2024; Kidd and Nieto McAvoy, 2025; Puzio, 2023), this body of work remains focused almost entirely on bereavement following death. It is unlikely, however, that these technologies will remain confined to this context. They may soon extend to situations of non-death loss, characterized by profound and enduring changes in the condition of a living person that disrupt relational continuity and reciprocity (Boss, 2010; Harris, 2019; Yehene et al., 2021). The following section examines this possibility, exploring the distinctive characteristics of such losses and the potential implications of incorporating AI technologies within these contexts.

### Non-death loss

Grief is a biopsychosocial response to the loss of something of value to the individual, whether a person, a relationship, or a spiritual value or symbol imbued with emotional significance (Freud, 1917). Despite the breadth of this definition, death continues to be perceived as the dominant cause of mourning within theoretical, research, and clinical discourse. In recent years, however, increasing recognition has been given to the fact that other life-altering events not necessarily involving death may also disrupt individuals’ relationships with significant others in ways that give rise to profound grief. Such grief may develop in ongoing situations characterized by uncertainty and the absence of clear closure. These circumstances generate a complex experience of continuing loss that does not converge into a single event and does not allow for definitive resolution (Yehene et al., 2021).

Various theorists have emphasized the psychological complexity inherent in such situations (for example, Doka, 2008; Harris, 2019; Rando, 1986; Roos, 2014; Bruce and Schultz, 2001; Singer et al., 2022). Particularly relevant to the present discussion is the concept of *ambiguous loss* articulated by Paulin Boss. This concept refers to situations in which there is a discrepancy between physical and psychological presence. In some cases, the person is psychologically present but physically absent, such as in situations of abduction, prolonged disappearance, or captivity. Conversely, there are circumstances in which the person is physically present but psychologically absent, as in cases of traumatic brain injury, stroke, severe psychiatric illness, persistent disorders of consciousness, or neurodegenerative diseases such as dementia (Boss, 2010).

Although both loss due to death and non-death loss entail processes of mourning, these processes differ in their characteristics and psychological dynamics. Table 1 presents a comparative analysis of the principal distinctions between these types of loss, with particular focus on dementia as a paradigmatic example of ongoing loss not resulting from death.

**TABLE 1:** Comparative analysis of the main distinctions between loss due to death and loss in dementia.

​	Death	Non-death loss
Finality	A single, finite loss event	Multiple cumulative losses without a clearly defined endpoint; characterized by progressive deterioration
Clarity	A certain and unambiguous situation	Ambiguity and indeterminate boundaries
Course of Grief	Gradual processing that may facilitate acceptance and closure	Ongoing, cyclical, and at times chronic process; feelings of stagnation and lack of integration
Cultural and Religious Framing	Presence of rituals, traditions, and mourning practices that provide structure and mark a boundary between life and death	Absence of clear concluding rituals; no definitive symbolic transitional moment
Social Recognition	Socially recognized and legitimate grief that typically elicits support	Disenfranchised or insufficiently recognized grief
Stigma	Relatively uncommon; may arise in specific circumstances such as socially prohibited relationships, suicide, overdose, or criminal involvement	More prevalent; often accompanied by shame and concealment
Identity and Roles	Clear status change, for example, widow, orphan, bereaved parent; adaptation to life without the deceased	Assumption of the primary caregiver role, often involving significant economic, physical, cognitive, and emotional burden and burnout
Memory of the Lost Person	Reliance on internalized representations, a relatively “closed system”	Difficulty consolidating a stable internal representation due to dissonance between the person’s current condition and their premorbid identity, constituting a relatively “open system”
Dominant Emotions	Profound sadness, longing, and yearning	Profound sadness and longing accompanied by confusion and ambivalence
Risk of Prolonged Grief Disorder	Generally low in the general population; elevated in specific contexts, such as traumatic or sudden death, or the death of a child	High risk, which may be further compounded by additional vulnerability factors

Although grief in situations of non-death loss is often a normative response to the complex reality of mourning loved ones who are still alive, a substantial subset of individuals experience intense reactions that lead to significant distress and functional impairment, thereby requiring professional intervention (e.g., Crawley et al., 2023; Manevich et al., 2023). In this context, a large scale meta-analysis and critical literature review found that among the various variables significantly predicting PGD (including depression, violent or sudden death, low education and income, female gender, anxious attachment style, and the loss of a child or partner), pre-death grief was the factor that contributed most strongly to the prediction of PGD (Buur et al., 2024). This finding underscores the vulnerability inherent in prolonged and ambiguous forms of loss, suggesting that individuals already engaged in ongoing grief processes may be particularly susceptible to subsequent complications.

The experience of loss not resulting from death has been examined in diverse clinical contexts, including terminal malignant illness (Treml et al., 2021), persistent disorders of consciousness (Guarnerio et al., 2012; Zaksh et al., 2019), and traumatic brain injury (Buckland et al., 2021; Thøgersen and Glintborg, 2022). However, given its scope and far-reaching consequences, dementia constitutes the most extensively studied paradigmatic case. The following discussion therefore focuses on loss in the context of dementia, though the central principles that emerge may also apply to other forms of ongoing non-death loss.

### Dementia as a paradigmatic case of non-death loss

Dementia (major neurocognitive disorder) is an umbrella term referring to a range of neurodegenerative syndromes characterized by significant cognitive decline relative to a previous level of functioning, in a manner that interferes with independence in daily life (APA, 2022; World Health Organization, 2021). Dementia constitutes a global health crisis with far-reaching consequences for healthcare systems, society at large, and informal support networks, which bear most of the caregiving burden (Alzheimer’s Association, 2025). Beyond its functional implications, dementia is marked by a gradual process of “psychological death” that is not synchronized with physical death (Boss, 2010).

GenAI technologies are increasingly being used as a supportive tool for informal caregivers of individuals living with dementia. These applications include the provision of tailored information, assistance in decision-making processes, emotional support, and monitoring of burden and burnout (Borna et al., 2024). However, to the best of the authors’ knowledge, no documented attempt has been made to integrate such technologies for the direct purpose of assisting family members in processing the distinctive grief associated with this form of ongoing loss.

Most existing applications focus on managing the daily challenges of caregiving and are not designed to create a virtual representation of the person’s premorbid personality, akin to the griefbots discussed in the previous section. To facilitate this examination, and in the absence of any existing technology designed specifically for this purpose, this article proposes a hypothetical application: the *PremorbidBot* (PMB), a chatbot designed to simulate the personality of a living person as they were prior to the onset of illness, drawing on digital materials such as correspondence, social media posts, recordings, and personal narratives. Unlike existing griefbots, such a system would operate while the person is still alive and receiving care.

Clinicians and scholars bear responsibility to prepare for a thoughtful examination of the potential implications of such technologies. Moreover, the possibility cannot be excluded that informal use of these technologies for such purposes is already occurring, while empirical research and regulatory frameworks have yet to catch up with the pace of development (Manevich and Aluma, 2025).

Unlike existing grief technologies, the PMB constitutes a distinct ethical category for three reasons. First, unlike griefbots of the deceased, the PMB operates while the represented individual is still alive, retaining legal rights and ongoing relational ties. Second, unlike caregiver-support AI tools (e.g., informational chatbots for dementia management), the PMB does not aim to provide practical guidance but rather to simulate the person’s premorbid personality and relational presence. Third, unlike general-purpose relational AI (companion chatbots such as *Replika*), the PMB is grounded in a specific individual’s personal data and is designed to represent that individual’s unique identity. These distinctions are summarized in Table 2.

**TABLE 2:** Distinguishing the PremorbidBot (PMB) from related AI technologies.

Dimension	Griefbot	Caregiver-support AI	Relational AI	PremorbidBot (PMB)
Status of represented person	Deceased	N/A (no specific person)	N/A (fictional persona)	Alive, with ongoing changes
Primary function	Memorialization; continuing bond	Information, decision support, burden monitoring	Companionship; emotional support	Simulation of premorbid personality for grief processing
Data source	Personal data of the deceased	General medical/caregiving knowledge	Generic or user-trained persona	Personal data of a living individual
Consent complexity	Retrospective (estate/family)	Standard user consent	Standard user consent	Dual: living person + caregiver; capacity questions
Coexistence with living person	No	Yes, but no simulation of the person	No (fictional entity)	Yes — simulation runs alongside living person
Risk of relational displacement	Low (no living counterpart)	Low (not relational)	Moderate (may replace human connection)	High (may replace engagement with the living person)

## Discussion

### An ethical framework for the PremorbidBot

Given that mourning processes occur both following death and in response to non-death loss, the ethical dilemmas associated with the integration of GenAI into coping are likely to share significant similarities. At the same time, important differences emerge when the person being simulated is still alive. The following discussion develops the distinct ethical considerations raised by the *PremorbidBot (PMB)* concept, focusing on its implications for caregivers of individuals living with dementia. While dementia serves here as the primary case of analysis, the ethical issues identified may extend to other forms of non-death loss in which GenAI-based simulations of a living person’s former self could be considered.

The growing number of ethical frameworks applied to GenAI in healthcare may lead to interpretive gaps and conceptual inconsistency (Gorelik et al., 2025). Accordingly, the present analysis is grounded in the four classical principles of biomedical ethics as articulated by Beauchamp and Childress (1979): respect for autonomy, beneficence, nonmaleficence, and justice.^1^

In the context of PMB use, applying the four principles requires a dual focus. The ethical analysis concerns not a single subject but a dyadic relational system between the informal caregiver and the person living with dementia. Each principle is therefore examined in relation both to the individual represented through the technology and to the active user of the system, with particular attention to nonmaleficence toward the more vulnerable party in the relationship. The purpose of this discussion is not to provide practical guidelines or to adopt a definitive stance for or against such technologies. Rather, it aims to identify core ethical questions that warrant careful examination in anticipation of future developments.

### Respect for autonomy

The principle of autonomy concerns respect for individual choice, informed consent, and self-determination. While questions of informed consent in AI-mediated grief have been raised in the post-death literature, the specific complications of dual consent in the PMB context, where one party may lack cognitive capacity and the other acts under significant distress, remain largely unexamined.

From the perspective of the person living with dementia, autonomy centers primarily on informed consent. Did the individual consent to the use of their image, voice, or correspondence for the creation of a digital representation? Even if explicit consent or prior directives were provided at an earlier stage, for example, in the form of a “digital will”, it remains necessary to assess whether such consent remains valid, given technological capabilities that may not have been foreseeable at the time of consent. The question of cognitive competence at the time of consent is especially salient. Beyond formal consent, it must be asked whether the use of a PMB respects the individual’s prior identity, values, and principles, and whether it safeguards their present dignity.

From the caregiver’s perspective, autonomy entails the right to determine how one copes with grief. However, this freedom may be compromised when decisions are made in a state of psychological vulnerability and instability. This raises the question of whether informed consent given under profound distress truly reflects a well-considered choice, particularly when the technology may have potentially irreversible effects on the grieving process. In addition, the opacity characteristic of many GenAI systems, which rely on complex processes not readily accessible to user understanding, complicates the possibility of fully informed decision-making.

Another autonomy-related concern involves the influence of a PMB on medical and caregiving decisions, such as placement in long-term care, hiring professional caregivers, or even more extreme actions grounded in statements attributed to the bot, for example, a simulated request to “end suffering”. Unlike griefbots of the deceased, such attributes concern a person who is still alive and whose present wishes, however difficult to ascertain, may differ from those expressed by the simulation. In such cases, there is a risk of undermining the caregiver’s independent judgment and of even potentially privileging the simulated voice of the person’s former self over their present needs and expressions.

### Beneficence

The principle of beneficence focuses on promoting the welfare and best interests of individuals. Preliminary evidence supports the potential of griefbots as supportive tools in post-death bereavement (Xygkou et al., 2023); however, their application to ongoing non-death loss, and the specific question of whether a PMB can facilitate grief processing while the person remains alive, are as yet untested.

With respect to the person living with dementia, it must be asked whether the use of a PMB advances their interests and whether any benefit can reasonably be expected. The benefit, if present, is likely indirect rather than direct, insofar as the technology may contribute to the caregiver’s psychological stability and functioning. At the same time, the patient’s interests might indirectly be served through the preservation and honoring of their premorbid identity. A simulation grounded in the person’s prior values, preferences, and modes of expression could help caregivers better understand and attend to their needs, effectively amplifying a voice that illness has progressively silenced.

For the caregiver, the technology may serve as a source of comfort, meaning, and emotional sustenance, facilitating the processing of grief-related emotions, reducing loneliness, alleviating symptoms, and improving functioning. Drawing on the theoretical frameworks discussed earlier, the PMB may function as a transitional object (Winnicott, 1971) — not in the aftermath of death, but in the midst of ongoing loss, mediating between the person as they were and as they are now. It may also support a form of continuing bond with the person’s premorbid self, enabling caregivers to preserve a sense of relational continuity even as the lived relationship undergoes profound change. Moreover, given that grief in non-death loss is frequently disenfranchised, socially unrecognized and unsupported, the very act of engaging with a PMB may serve a validating function, legitimizing an experience of loss that is often invisible to others.

At the broader family level, a PMB may also facilitate shared understanding among family members by providing a common reference point for who the person was, thereby supporting more cohesive caregiving and decision-making. However, it may equally generate confusion and disagreement, particularly when family members interpret the simulation’s outputs differently or when the bot’s responses conflict with the person’s current expressions and behavior. Drawing on the concept of the “artificial third” (Haber et al., 2024), it must be recognized that a PMB introduces a new entity into the caregiving dynamic—one that simulates a member of the family system itself. The extent to which this presence enhances family collaboration or disrupts existing relational equilibria remains an open question that warrants empirical investigation.

### Nonmaleficence

The principle of nonmaleficence emphasizes the prevention of physical, psychological, moral, or social harm and the mitigation of potential risks. Documented concerns about emotional dependency and disrupted grief processing in post-death griefbot use (e.g., Kawashima et al., 2023) provide a grounded basis for anticipating analogous risks here, though the distinct possibility that a PMB may lead caregivers to prefer the simulation over the living person represents a novel and as yet hypothetical concern.

In relation to the person living with dementia, potential harms include violations of dignity, reputation, and legacy through distortion of their persona, whether by presenting a unidimensional representation or through algorithmic “hallucinations”, i.e., fabricated outputs that may be mistakenly attributed to the person’s actual views or character. There is also risk of exposure or leakage of sensitive personal data and use of such data contrary to the individual’s wishes. GenAI platforms collect substantial amounts of personal information, creating tangible risks of security breaches, misuse of data, and commercial exploitation without transparency (Elyoseph et al., 2024).

From the caregiver’s perspective, PMB use may exacerbate psychological distress, foster emotional dependency, contribute to social withdrawal, or interfere with caregiving responsibilities. A particular risk unique to the PMB context is that the caregiver may come to prefer interaction with the simulation over engagement with the person as they are now, thereby deepening the relational rupture that the technology was intended to address. Algorithmic errors or biases may result in inaccurate or harmful recommendations. In addition, a “secondary loss” may occur in the event of technical failure, service policy changes, or platform closure, effectively replicating the original experience of loss and potentially retraumatizing the caregiver.

### Justice

The principle of justice concerns fairness, equitable distribution of rights and resources, and institutional accountability. Questions of data ownership and commercial exploitation are well established in the broader AI ethics literature; their specific manifestation in the PMB context, where the simulated individual is alive and may retain active legal rights over their digital representation, constitutes a plausible but as yet unexamined extension.

From the perspective of the person living with dementia, it is necessary to examine whether their data and digital identity are exploited for commercial purposes beyond the original intent for which they were provided. Questions arise regarding ownership and control of digital persona and associated data. Specifically, it remains unclear whether ownership resides with the technology company, the person whose identity is being simulated, or the caregiver who provided the materials. Unlike in the case of griefbots for the deceased, the simulated individual is still alive and may retain, directly or through legal guardianship, active rights over their digital representation. These issues become especially acute if the service is discontinued or the company ceases operation, raising questions of responsibility in cases of data loss or emotional harm.

From the caregiver’s perspective, it must be asked whether vulnerable populations are being exploited through the commercialization of emotional distress. Business models driven by financial incentives may encourage prolonged use and emotional dependency, rather than facilitating adaptation to loss. Additionally, consideration must be given to equitable access. If such technologies are not universally accessible, they may create new digital hierarchies in grieving practices and thereby deepen social inequality.

In light of these considerations, the implementation of PMB in the context of dementia-related grief requires a cautious and multidimensional ethical evaluation that balances the rights, benefits, and risks affecting both parties within a relationally structured and vulnerable situation.

## Conclusion and future directions

This article has examined the ethical implications of extending GenAI-based grief technologies beyond bereavement after death into the domain of non-death loss. Through the introduction of the *PremorbidBot (PMB)* concept and its analysis via the four principles of biomedical ethics (Beauchamp and Childress 1979), significant ethical issues have been identified that arise when technologies originally intended for memorialization extend into the terrain of ambiguous loss (see Table 3 for a summary). The resulting ethical challenge is distinctive, as the individual represented through technology continues to live alongside their digital representation concurrently, creating a complex overlap between digital representation and a living, changing, and vulnerable existence.

**TABLE 3:** Ethical analysis of the PremorbidBot (PMB): Four principles of biomedical ethics applied to the caregiving dyad and the PMB.

Principle	Person living with dementia	Informal caregiver	PremorbidBot as an artificial third core issues	Key questions
Respect for Autonomy	• Informed consent for creation of digital representation may not have been given • Prior consent may lose validity given unforeseen technological developments • Cognitive competence at time of consent is questionable • Risk of violating prior identity, values, and present dignity • Concept of a “digital will” raises unresolved questions of capacity and foresight	• Right to determine how to cope with grief and loss • Consent under profound psychological distress may not reflect autonomous choice • Opacity of GenAI systems limits fully informed decision-making • Simulated voice of former self may override attention to person’s present needs and expressions • Bot outputs may influence medical and caregiving decisions	• Simulates agency without possessing it, yet outputs are received as reflecting the will of another • Neither autonomous nor transparent in its generative processes • May attribute preferences and intentions to a living person without verification • Creates an illusion of dialogue where no genuine intersubjectivity exists	• Can meaningful informed consent be obtained from a person with progressive cognitive decline? • Does engaging with a PremorbidBot constitute an autonomous coping choice or a vulnerability-driven response? • How should attributed agency be distinguished from genuine self-expression?
Beneficence	• Indirect benefit through enhanced caregiver psychological stability and capacity • Preservation and honoring of premorbid identity • Amplifying a voice progressively silenced by illness • Helping caregivers better understand and attend to current needs	• Source of emotional comfort, meaning, and sustenance • May function as a transitional object in ongoing loss (Winnicott, 1971) • Supporting continuing bond with the person’s premorbid self • Legitimizing disenfranchised grief in non-death loss (Doka, 2008) • Possible reduction of prolonged grief disorder risk (Buur et al., 2024) • Shared reference point for family understanding and cohesive caregiving	• May serve as a mediating bridge within the caregiving dyad • Could facilitate family communication through a shared frame of reference • Risk of becoming a substitute for direct engagement with the living person • May generate confusion when bot outputs conflict with current behavior or needs • Introduction of an “artificial third” (Haber et al., 2024) may enhance collaboration or disrupt relational equilibria	• Does the PremorbidBot enhance the caregiving relationship or create a parallel substitute? • To what extent does the artificial third become central at the expense of the living person? • Can the bot facilitate family cohesion, or does it risk deepening disagreements over care?
Non- maleficence	• Distortion of persona through unidimensional or inaccurate representation • Algorithmic hallucinations mistakenly attributed to the person’s actual views or character • Exposure or leakage of sensitive personal data • Commercial exploitation of personal information without transparency or consent • Violation of dignity through a flattened, static digital depiction of a dynamic individual	• Exacerbation of psychological distress and social withdrawal • Emotional dependency — preferring the simulation over the living person, deepening relational rupture • Interference with caregiving responsibilities and real-world engagement • Secondary loss from platform closure or technical failure, replicating and potentially retraumatizing the original experience of loss	• Generates outputs that may carry no relation to the person’s actual identity or wishes • Lacks capacity for self-correction; errors compound without internal accountability • May become a locus of emotional attachment, displacing the living person in the relational system • Platform-dependent: its existence is contingent on commercial viability, not relational need	• How can algorithmic hallucinations be prevented from distorting a living person’s represented identity? • What safeguards can mitigate the risk of emotional dependency and caregiver withdrawal from the living person? • Who bears responsibility for harm caused by inaccurate or misleading bot outputs?
Justice	• Data exploitation for commercial purposes beyond original intent • Unclear ownership of digital persona and associated data — company, caregiver, or living person • Unlike griefbots for deceased persons, the simulated individual is alive and may retain active legal rights over their digital representation • Responsibility gaps if service is discontinued or company ceases operation	• Commercialization of emotional distress in vulnerable populations • Business models encouraging prolonged dependency rather than facilitating adaptation • Unequal access creating new digital hierarchies in grieving practices • Deepening social inequality through uneven availability of technology	• Exists within a commercial ecosystem with interests potentially misaligned with both parties • Data collected to construct the bot may be repurposed beyond the relational context • No regulatory framework currently governs the creation or use of digital simulations of living persons • Raises unprecedented questions of digital personhood and representational rights	• Who holds ownership of the digital persona — the company, the caregiver, or the living person? • How can regulatory frameworks ensure protection of both parties without stifling beneficial innovation? • What obligations do platform providers have when discontinuing a service on which users have become emotionally dependent?

Analysis draws on Beauchamp and Childress (1979) four principles of biomedical ethics, applied to the dyadic relational system of the person living with dementia and their informal caregiver, with the PremorbidBot examined as an artificial third (Haber et al., 2024) that enters and reshapes the caregiving dynamic.

As a preliminary step toward ethical evaluation and decision-making in AI-mediated non-death loss contexts, we propose the ALIVE framework (Table 4). The framework identifies five interrelated conditions for ethically responsible development and use: Autonomy and Consent (dual informed consent with attention to capacity and vulnerability), Living Presence (recognition that the simulated individual coexists with their digital representation), Intended Benefit (demonstrable contribution to the wellbeing of both parties in the dyad), Vigilance Against Harm (ongoing monitoring for dependency, relational displacement, and identity distortion), and Equity and Accountability (fair access, data protection, and institutional responsibility). The Living Presence condition, in particular, captures what distinguishes the PMB from post-death grief technologies and underscores why existing ethical frameworks require extension.

**TABLE 4:** The ALIVE Framework for Ethical Evaluation of PremorbidBot (PMB) Technologies: A preliminary model for responsible development and use.

​	Principle	Core question	Practical implication
A	Autonomy & Consent	Has dual informed consent been obtained — from the represented person (or their surrogate) and from the caregiver — with attention to capacity and vulnerability?	PMB use should be restricted when the represented individual has not provided prior consent and no valid surrogate mechanism exists. Consent must be periodically reassessed as cognitive and relational conditions evolve
L	Living Presence	Does the framework account for the fact that the simulated individual is alive, changing, and coexists with their digital representation?	System design must prioritize engagement with the living person over interaction with the simulation. Features that encourage prolonged or exclusive use at the expense of direct caregiving contact should be avoided
I	Intended Benefit	Is there demonstrable benefit to the wellbeing of both parties in the caregiving dyad?	Deployment should be accompanied by clinical assessment of benefit. In the absence of evidence for positive outcomes, use should be considered experimental and subject to professional oversight
V	Vigilance Against Harm	Are safeguards in place to monitor for emotional dependency, relational displacement, and identity distortion?	PMB use should be suspended when clinical indicators suggest relational displacement or grief pathology exacerbation. Built-in safeguards — usage monitoring, transparency disclosures, clinical escalation triggers — should be treated as design requirements
E	Equity & Accountability	Is access equitable, data protected, and institutional responsibility clearly assigned?	Platforms must ensure data ownership resides with the represented individual or their legal guardian, not the service provider. Business models incentivizing prolonged emotional dependency should be regarded as ethically impermissible. Contingency protocols for service discontinuation are required

The ALIVE, framework is offered as a conceptual foundation for future empirical and normative inquiry. The cumulative satisfaction of all five conditions is proposed as a necessary precondition for ethically responsible PMB use.

At this stage, we suggest that the cumulative satisfaction of all five ALIVE conditions be considered a necessary precondition for ethically responsible use. When one or more conditions cannot be met, particularly those of informed consent and vigilance against harm, the deployment of PMB technologies should be approached with significant caution or withheld until adequate safeguards are in place.

Although the analysis has centered on dementia, the ethical considerations identified are also relevant to other contexts characterized by ongoing and ambiguous loss, such as families of abducted or missing persons, individuals in comatose states, traumatic brain injury, or severe psychiatric illness. In each of these cases, a persistent gap exists between presence and absence, potentially motivating the use of technological simulations to navigate the tension between connection and separation. Future research should examine whether and how the ethical framework proposed here applies to these diverse contexts, each of which presents its own relational dynamics and vulnerabilities.

More broadly, the rapid advancement of GenAI technologies now enables the creation of digital simulations of real individuals with remarkable ease, through voice cloning, personality modeling, and conversational replication. This accessibility means that the ethical questions raised in this article are not confined to clinical contexts alone. Wherever a significant relationship is disrupted or lost, whether through illness, disappearance, or even the dissolution of a romantic or close friendship, the possibility of generating an artificial version of the absent other is increasingly within reach. The ethical framework proposed here may therefore serve as a foundation for examining a broader range of situations in which individuals turn to GenAI as a means of coping with relational loss.

Looking ahead, digital representations may extend beyond virtual environments and acquire more tangible forms, potentially taking the form of physical embodiments capable of touch, embrace, or simulated presence. As these technologies evolve, the ethical questions identified in this article will only grow in urgency and complexity. While the trajectory of technological development cannot be predicted with certainty, ethical responsibility demands anticipatory reflection and careful examination. The framework proposed here offers a starting point for the anticipatory reflection required for the responsible development of such technologies. Ultimately, the challenge is not merely technological but deeply human: how to honor the bond between caregiver and loved one when the boundaries between presence and absence, memory and simulation, the person who was and the person who remains, are no longer clear.

## Data Availability

The original contributions presented in the study are included in the article/supplementary material, further inquiries can be directed to the corresponding author.

## Footnotes

1 It should be emphasized that the issue entails inherent complexity; accordingly, the division into four principles cannot be entirely discrete or hermetically sealed. In practice, substantial overlap exists among the issues, such that a particular concern may be relevant to more than one principle simultaneously and may be examined from different perspectives within the same case. Nevertheless, for the sake of analytical clarity, each discussion seeks to foreground the aspect most directly related to the principle under consideration, while acknowledging the interconnections and potential tensions among the principles.

## References

[B1] Alzheimer's Association (2025). 2025 alzheimer's disease facts and figures. Alzheimer's and Dementia. Available online at: https://www.alz.org/getmedia/ef8f48f9-ad36-48ea-87f9-b74034635c1e/alzheimers-facts-and-figures.pdf (Accessed February 1, 2026). 10.1016/j.jalz.2012.02.001 22404854

[B2] American Psychiatric Association (APA) (2022). Diagnostic and statistical manual of mental disorders. 5th ed.

[B3] Beauchamp T. L. Childress J. F. (1979). Principles of biomedical ethics (8th ed). Oxford University Press.

[B4] Bhatt S. (2025). Digital mental health: role of artificial intelligence in psychotherapy. Ann. Neurosci. 32 (2), 117–127. 10.1177/09727531231221612 39544658 PMC11559931

[B5] Blease C. Rodman A. (2024). Generative artificial intelligence in mental healthcare: an ethical evaluation. Curr. Treat. Options Psychiatry 12 (1), 1–9. 10.1007/s40501-024-00340-x

[B6] Borna S. Maniaci M. J. Haider C. R. Gomez-Cabello C. A. Pressman S. M. Haider S. A. (2024). Artificial intelligence support for informal patient caregivers: a systematic review. Bioengineering 11 (5), 483. 10.3390/bioengineering11050483 38790350 PMC11118398

[B7] Boss P. (2010). The trauma and complicated grief of ambiguous loss. Pastor. Psychol. 59 (2), 137–145. 10.1007/s11089-009-0264-0

[B8] Brescó de Luna I. Jiménez-Alonso B. (2024). Deathbots: discussing the use of Artificial Intelligence in grief. Stud. Psychol. 45 (1), 103122. 10.1177/02109395241241387

[B9] Bruce E. J. Schultz C. L. (2001). Nonfinite loss and grief: a psychoeducational approach. Baltimore, MD: Paul H Brookes Publishing.

[B10] Buckland S. Kaminskiy E. Bright P. (2021). Individual and family experiences of loss after acquired brain injury: a multi-method investigation. Neuropsychol. Rehabil. 31 (4), 531–551. 10.1080/09602011.2019.1708415 31902308

[B11] Buur C. Zachariae R. Komischke-Konnerup K. B. Marello M. M. Schierff L. H. O’Connor M. (2024). Risk factors for prolonged grief symptoms: a systematic review and meta-analysis. Clin. Psychol. Rev. 107, 102375. 10.1016/j.cpr.2023.102375 38181586

[B12] Campbell S. M. Liu P. Nyholm S. (2025). Can chatbots preserve our relationships with the dead? J. Am. Philosophical Assoc. 11 (2), 1–19. 10.1017/apa.2025.1

[B13] Christensen D. R. Sumiala J. (2024). Introducing the special issue digital death: transforming rituals, history, and the afterlife. Soc. Sci. 13 (7), 346. 10.3390/socsci13070346

[B14] Crawley S. Sampson E. L. Moore K. J. Kupeli N. West E. (2023). Grief in family carers of people living with dementia: a systematic review. Int. Psychogeriatrics 35, 1–32. 10.1017/S1041610221002787 35086600

[B15] Cross S. Bell I. Nicholas J. Valentine L. Mangelsdorf S. Baker S. (2024). Use of AI in mental health care: community and mental health professionals survey. JMIR Ment. Health 11, e60589. 10.2196/60589 39392869 PMC11488652

[B16] Cruz-Gonzalez P. He A. W.-J. Lam E. P. Ng I. M. C. Li M. W. Hou R. (2025). Artificial intelligence in mental health care: a systematic review of diagnosis, monitoring, and intervention applications. Psychol. Med. 55 (e18), 1–52. 10.1017/S0033291724003295 39911020 PMC12017374

[B17] Degni F. (2025). “The afterlife in the age of AI: a psychological, ethical, and technological analysis,” in Ethical and technological analysis. 10.2139/ssrn.5183433

[B18] Doka K. J. (2008). “Disenfranchised grief in historical and cultural perspective,” in Handbook of bereavement research and practice: advances in theory and intervention. Editors Stroebe M. S. Hansson R. O. Schut H. Stroebe W. (Washington, DC: American Psychological Association), 223–240.

[B19] Dwi M. (2025). Ethical and psychological implications of Generative AI in digital afterlife technologies: a systematic literature review on responsible inclusive innovation. J. Responsible Technol. 24, 100136. 10.1016/j.jrt.2025.100136

[B20] Elder A. M. (2019). Conversation from beyond the grave? A Neo-Confucian ethics of chatbots of the dead. J. Appl. Philosophy 37 (1), 73–88. 10.1111/japp.12369

[B21] Elyoseph Z. Tal A. Haber Y. Angert T. Gur T. Simon T. (2024). An ethical perspective on the democratization of mental health with generative artificial intelligence. JMIR Ment. Health 11, e58011. 10.2196/58011 39417792 PMC11500620

[B22] Fabry R. E. Alfano M. (2024). The affective scaffolding of grief in the digital age: the case of deathbots. Topoi 43, 757–769. 10.1007/s11245-023-09995-2

[B23] Figueroa-Torres M. (2024). Affection as a service: ghostbots and the changing nature of mourning. Comput. Law and Secur. Rev. 52, 105943. 10.1016/j.clsr.2024.105943

[B24] Freud S. (1917). Mourning and melancholia. Standard edition of the complete psychological works of Sigmund Freud. London, UK: Hogarth.

[B25] Fu K. Ye C. Wang Z. Wu M. Liu Z. Yuan Y. (2025). Ethical dilemmas and the reconstruction of subjectivity in digital mourning in the age of AI: an empirical study on the acceptance intentions of bereaved family members of cancer patients. Front. Digital Health 7, 1618169. 10.3389/fdgth.2025.1618169 40692654 PMC12278142

[B26] Gilat Y. (2025). Is artificial intelligence the “future shock” of mental health? Haaretz. Available online at: https://www.haaretz.co.il/health/2025-01-29/ty-article-magazine/.premium/00000194-ac71-dbc9-a59f-ac79a5080000 (Accessed February 1, 2026).

[B27] Gorelik A. J. Li M. Hahne J. Wang J. Ren Y. Yang L. (2025). Ethics of AI in healthcare: a scoping review demonstrating applicability of a foundational framework. Front. Digital Health 7, 1662642. 10.3389/fdgth.2025.1662642 41000336 PMC12459206

[B28] Grandinetti J. DeAtley T. Bruinsma J. (2020). “The dead speak: big data and digitally mediated death,” in *AoIR selected papers of internet research,* 2020. 10.5210/spir.v2020i0.11122

[B29] Guarnerio C. Prunas A. Della Fontana I. Chiambretto P. (2012). Prevalence and comorbidity of prolonged grief disorder in a sample of caregivers of patients in a vegetative state. Psychiatr. Q. 83 (1), 65–73. 10.1007/s11126-011-9183-1 21691850

[B30] Haber Y. Levkovich I. Hadar-Shoval D. Elyoseph Z. (2024). The artificial third: a broad view of the effects of introducing Generative Artificial Intelligence on psychotherapy. JMIR Ment. Health 11, e54781. 10.2196/54781 38787297 PMC11137430

[B31] Haber Y. Hadar Shoval D. Levkovich I. Yinon D. Gigi K. Pen O. (2025). The externalization of internal experiences in psychotherapy through generative artificial intelligence: a theoretical, clinical, and ethical analysis. Front. Digital Health 7, 1512273. 10.3389/fdgth.2025.1512273 39968063 PMC11832678

[B32] Harris D. L. (2019). Non-death loss and grief: context and clinical implications (New York, NY: Routledge).

[B33] Hollanek T. Nowaczyk-Basińska K. (2024). Griefbots, deadbots, postmortem avatars: on responsible applications of Generative AI in the digital afterlife industry. Philosophy and Technol. 37, 63. 10.1007/s13347-024-00744-w

[B34] Hurtado Hurtado J. (2023). Towards a postmortal society of virtualised ancestors? The virtual deceased person and the preservation of the social bond. Mortality 28 (1), 90–105. 10.1080/13576275.2021.1878349

[B35] Kakarala S. E. Roberts K. E. Rogers M. Coats T. Falzarano F. Gang J. (2020). The neurobiological reward system in Prolonged Grief Disorder (PGD): a systematic review. Psychiatry Res. Neuroimaging 303, 111135. 10.1016/j.pscychresns.2020.111135 32629197 PMC7442719

[B36] Kawashima D. Kempe T. Koga Y. (2023). “I want my loved one back virtually”: exploring the desire of bereaved people to create and maintain digital bonds with their deceased loved ones. OMEGA–Journal Death Dying 0 (0), 1049–1065. 10.1177/00302228231194857 37584392

[B37] Kidd J. Nieto McAvoy E. (2025). “Synthetic afterlives: deathbots as affective infrastructures of memory,” Mem. Mind Media. 4. 10.1017/mem.2025.10013

[B38] Klass D. Steffen E. M. (2018). Continuing bonds in bereavement: new directions for research and practice. New York: Routledge.

[B39] Krueger J. Osler L. (2022). Communing with the dead online: Chatbots, grief, and continuing bonds. J. Conscious. Stud. 29 (9–10), 222–252. 10.53765/20512201.29.9.222

[B40] Manevich A. Aluma S. (2025). Artificial continuing bonds: a proposed framework for responsible integration of generative artificial intelligence in clinical thanatology. OMEGA – J. Death Dying, 302228251372418. 10.1177/00302228251372418 40854560

[B41] Manevich A. Yehene E. Rubin S. S. (2023). A case for inclusion of disordered non-death interpersonal grief as an official diagnosis: rationale, challenges and opportunities. Front. Psychiatry 14, 1300565. 10.3389/fpsyt.2023.1300565 38161721 PMC10757611

[B42] Manevich A. Lahad M. Rubin S. S. Shalev R. Shalev D. Ablin E. (2025). “Machine mediated grief”: a position paper by an expert panel on the integration of artificial intelligence in the fields of loss and bereavement. Psychoactuallya - Isr. Psychol. Assoc. (100), 27–33. Available online at: https://www.psychology.org.il/staticcontent/magazine1225/index.html (Accessed February 1, 2026).

[B43] Morris M. R. Brubaker J. R. (2025). “Generative ghosts: anticipating benefits and risks of AI afterlives,” in In *proceedings of the 2025 CHI conference on human factors in computing systems* , 1–14. 10.1145/3706598.3713758

[B44] Olawade D. B. Wada O. Z. Odetayo A. David-Olawade A. C. Asaolu F. Eberhardt J. (2024). Enhancing mental health with Artificial Intelligence: current trends and future prospects. J. Med. Surg. Public Health 3, 100099. 10.1016/j.glmedi.2024.100099

[B45] O’Connor M. (2024). Grief universalism: a perennial problem pattern returning in digital grief studies? Soc. Sci. 13 (4), 208. 10.3390/socsci13040208

[B46] Pizzoli S. F. M. Vergani L. Monzani D. Scotto L. Cincidda C. Pravettoni G. (2024). The sound of grief: a critical discussion on the experience of creating and listening to the digitally reproduced voice of the deceased. OMEGA–Journal Death Dying 2218–2226. 10.1177/00302228231225273 PMC12891239 38176688

[B47] Puzio A. (2023). When the digital continues after death: ethical perspectives on death tech and the digital afterlife. Commun. Soc. 56 (3), 427–436. 10.5771/0010-3497-2023-3-427

[B48] Rando T. A. (1986). Loss and anticipatory grief. Lexington, MA: Lexington Books.

[B49] Recuber T. (2023). The digital departed: how we face death, commemorate life, and chase virtual immortality. New York, NY: NYU Press.

[B50] Roos S. (2014). Chronic sorrow: a living loss. New York, NY: Routledge.

[B51] Seckman C. (2025). The role of artificial intelligence in the grieving process: a literature review. J. Inf. Nurs. 10 (1), 7–20. 10.63123/JIN.2025.10.1.7

[B52] Singer J. Roberts K. E. McLean E. Fadalla C. Coats T. Rogers M. (2022). An examination and proposed definitions of family members’ grief prior to the death of individuals with a life-limiting illness: a systematic review. Palliat. Med. 36 (4), 581–608. 10.1177/02692163221074540 35196915 PMC10098140

[B53] Thøgersen C. M. S. Glintborg C. (2022). Ambiguous loss and disenfranchised grief among spouses of brain injury survivors. Nord. Psychol. 74 (1), 16–29. 10.1080/19012276.2020.1862699

[B54] Treml J. Schmidt V. Nagl M. Kersting A. (2021). Pre-loss grief and preparedness for death among caregivers of terminally ill cancer patients: a systematic review. Soc. Sci. and Med. 284, 114240. 10.1016/j.socscimed.2021.114240 34303292

[B55] Vallor S. (2024). The AI mirror: how to reclaim our humanity in an age of machine thinking. Oxford University Press.

[B56] Winnicott D. W. (1971). Playing and reality. London: Tavistock.

[B57] World Health Organization (WHO) (2021). International classification of diseases for mortality and morbidity statistics.

[B58] Xygkou A. Siriaraya P. Covaci A. Prigerson H. G. Neimeyer R. Ang C. S. (2023). “The “conversation” about loss: understanding how chatbot technology was used in supporting people in grief,” in *Pr*oceedings of the 2023 CHI conference on human factors in computing systems (CHI ’23) (Hamburg, Germany: ACM). 10.1145/3544548.3581154

[B59] Yang N. Khanna G. J. (2025). AI and technology in grief support: clinical implications and ethical considerations. Couns. Psychol. 53 (4), 579–604. 10.1177/00110000251352568

[B60] Yehene E. Manevich A. Rubin S. S. (2021). Caregivers’ grief in acquired Non-Death Interpersonal Loss (NoDIL): a process based model with implications for theory, research and intervention. Front. Psychol. 12, 676536. 10.3389/fpsyg.2021.676536 33995234 PMC8119762

[B61] Yockel M. R. Sleiman M. M. Jr. Doherty H. Adams R. Davis K. M. Groninger H. (2025). Mental health professionals’ views on artificial intelligence as an aide for children anticipating or suffering the loss of a parent to cancer: helpful or harmful? Children 12 (6), 763. 10.3390/children12060763 40564721 PMC12191389

[B62] Zaksh Y. Yehene E. Elyashiv M. Altman A. (2019). Partially dead, partially separated: establishing the mechanism between ambiguous loss and grief reaction among caregivers of patients with prolonged disorders of consciousness. Clin. Rehabil. 33 (2), 345–356. 10.1177/0269215518802339 30255716

